# Extremely Low-Frequency Magnetic Field Enhances the Therapeutic Efficacy of Low-Dose Cisplatin in the Treatment of Ehrlich Carcinoma

**DOI:** 10.1155/2013/189352

**Published:** 2013-01-14

**Authors:** Nihal S. El-Bialy, Monira M. Rageh

**Affiliations:** Biophysics Department, Faculty of Science, Cairo University, Al Gammaa Street, Giza 12613, Egypt

## Abstract

The present study examines the therapeutic efficacy of the administration of low-dose cisplatin (*cis*) followed by exposure to extremely low-frequency magnetic field (ELF-MF), with an average intensity of 10 mT, on Ehrlich carcinoma in vivo. The cytotoxic and genotoxic actions of this combination were studied using comet assay, mitotic index (MI), and the induction of micronucleus (MN). Moreover, the inhibition of tumor growth was also measured. Treatment with cisplatin and ELF-MF (group A) increased the number of damaged cells by 54% compared with 41% for mice treated with cisplatin alone (group B), 20% for mice treated by exposure to ELF-MF (group C), and 9% for the control group (group D). Also the mitotic index decreased significantly for all treated groups (*P* < 0.001). The decrement percent for the treated groups (A, B, and C) were 70%, 65%, and 22%, respectively, compared with the control group (D). Additionally, the rate of tumor growth at day 12 was suppressed significantly (*P* < 0.001) for groups A, B, and C with respect to group (D). These results suggest that ELF-MF enhanced the cytotoxic activity of cisplatin and potentiate the benefit of using a combination of low-dose cisplatin and ELF-MF in the treatment of Ehrlich carcinoma.

## 1. Introduction

Platinum-based chemotherapeutic regimens have been widely used against many human cancers including oral, lung, head and neck cancer, metastatic tumors of testis and ovaries and many other solid tumors [1, 2]. The anticancer activity of cisplatin comes from its interactions with DNA. The drug binds with N7 of purine bases forming monoadducts which are later transformed into inter and intrastrand cross links by reaction of second reactive site of the drug with the second nucleobase. Such cisplatin-DNA adducts can inhibit fundamental cellular processes including replication, transcription, translation, and DNA repair [3].

 Cisplatin must be used with a very high dose to maximize its antineoplastic effect. Such dose has been impeded by its sever toxicities, including nephrotoxicity, gastrointestinal toxicity, peripheral neuropathy, and ototoxicity [4–6]. The impairment of kidney function is considered as the main side effect of cisplatin, which is able to generate reactive oxygen species, such as superoxide anion and hydroxyl radical [7, 8]. Also nephrotoxicity is closely associated with an increase in lipid peroxidation in the kidney tissues [9]. Additionally, cisplatin-based chemotherapy induces a fall in patient plasma concentrations of various antioxidants [10]. This may lead to failure of the antioxidative defense mechanism against free-radical-mediated organ damage and genotoxicity. Accordingly, the significant risk of cisplatin frequently hinders its use with such effective dose. To address this problem, attention has been focused on finding a novel combination of anticancer agents with nonoverlapping mechanisms of action to achieve enhanced efficacy with decreased side effects. 

 Consequently, [11] reported the possible synergism between ELF-MF and chemotherapy, where a low dose of cisplatin was administrated followed by exposure to ELF-MF in order to reduce the drug side effects while keeping its therapeutic efficiency. The study hypothesized that static and extremely low frequency magnetic fields (ELF-MF) selectively act on cell signaling through their effects on charged matter motion.

 The influence of static and ELF-MF on tumor growth, apoptosis, and P53 immunohistochemical expression have been studied in a series of independent reports. Their results indicated that simultaneous use of static and extremely low frequency magnetic fields with an average intensity higher than 3.59 mT, significantly inhibited tumor growth, decreased tumor cell mitotic index, and lowered the proliferative activity. Moreover, an increase in apoptosis and a corresponding reduction of immunoreactive P53 expression were also observed [12–15]. 

 Therefore, the aim of the present work is to investigate the effectiveness of administration of low-dose cisplatin followed by exposure to ELF-MF, with an average intensity of 10 mT, on the growth of Ehrlich Carcinoma by studying cytotoxicity and DNA damage in tumor cells. 

## 2. Materials and Methods

### 2.1. Cell Culture and Tumor Inoculation

 Ehrlich ascites carcinoma cells (obtained from National Cancer Institute “NCI”, Cairo University) containing 1 × 10^6^ cells were intraperitoneally (i.p.) injected into female mice. Ascites fluid was collected on the 7th day after injection. The Ehrlich cells were washed twice and then resuspended in 0.09 saline (5 × 10^6^ viable cells). Female BALB mice (obtained from the animal house of NCI, with a body weight 22–25 g, 7-8 weeks old) were injected subcutaneously in their right flanks where the tumor was developed in a single and solid form. Tumor growth was monitored postinoculation until the desired volume was about 0.3 to 0.6 cm^3^. All animal procedures and care were performed using guidelines for the Care and Use of Laboratory Animals [16] and approved by animal Ethics Committee at Cairo University. 

### 2.2. Treatment Protocols

 The experiment was run on a total of 40 mice. Ten days after tumor cell inoculation, mice were randomly assigned to experimental groups. Mice of group (A) were treated three times on experimental days 1, 4, and 7 with 0.1 mL cisplatin (3 mg/kg i.p.) followed by exposure to 50 Hz, 10 mT ELF-MF, 1 hr daily for 2 weeks. Mice of group (B) were treated three times on experimental days 1, 4, and 7 with 0.1 mL cisplatin (3 mg/kg i.p.). Mice of group (C) were injected with 0.1 mL saline (instead of cisplatin) three times on experimental days 1, 4, and 7 followed by exposure to 50 Hz,10 mT ELF-MF, 1 hr daily for 2 weeks. Mice of group (D) were neither injected with cisplatin nor exposed to ELF-MF. During the treatment protocol, the tumor growth was monitored every three days over a period of 12 days for all the experimental groups A, B, C, and D. At the end of the treatment protocol, the mice of each group were divided so that 5 mice were sacrificed for the assessment of both comet and micronucleus and the other 5 mice were used to evaluate mitotic index. 

### 2.3. Magnetic Field Exposure

 The exposure was performed by a magnet with a fixed magnetic field value of 10 mT ±0.025. The magnetic field was generated by a solenoid carrying current of 18 A (ampere) at 50 Hz from the main supply (220–230 Volt) via a Variac (made in Yugoslavia). The magnet consisted of a coil with 320 turns made of electrically insulated 0.8 mm copper wire. The coil was wounded around a copper cylinder of 2 mm thickness, 40 cm diameter, and 40 cm length. The cylinder wall was earthed to eliminate the electric field. The magnetic field was measured at different locations to find out the most homogenous zone inside the solenoid core. This was done using Gauss/Tesla meter model 4048 with probe T-4048 manufactured by Bell Technologies Inc. (Orlando-Florida USA). Plastic cages containing groups (A) and (C) were placed in the middle of the exposure chamber prior to ELF-MF exposure. 

### 2.4. Comet Assay (Single Cell Gel Electrophoresis)

Comet assay (single cell gel electrophoresis) is considered as a rapid, simple, visual, and sensitive technique to assess DNA fragmentation typical for toxic DNA damage and early stage of apoptosis [17, 18]. The comet assay was performed under alkaline conditions (pH > 13) according to the method developed by Singh et al. [19] and Tice et al. [20]. Briefly, a small piece of tumor tissues (*n* = 5) from each group were placed in 1ml cold HBSS containing 20 mM EDTA (ethylenediaminetetraacetic acid)/10% DMSO (dimethylsulfoxide, Qualigens, CPW59). The tissues were minced into fine pieces and let settled. 5 *μ*L of aliquot was mixed with 70 *μ*L of 0.7% low melting point (LMP) agarose (Sigma, A9414). This agarose was prepared in Ca^2+^, Mg^2+^ free PBS (phosphate buffered saline, HiMedia, TS1006) at 37°C and placed on a microscope slide, which was already covered with a thin layer of 0.5% normal melting point (NMP) agarose (HiMedia.RM273). After cooling at 4°C for 5 min, slides were covered with a third layer of LMP agarose. After solidification at 4°C for 5 min, slides were immersed in freshly prepared cold lysis solution (2.5 M NaCl, 1 mM Na_2 _EDTA, 10 mM tris base, pH 10, with 1% Triton X-100 and 10% DMSO added just before use) at 4°C for at least 1 h. Following lyses, slides were placed in a horizontal gel electrophoresis unit and incubated in fresh alkaline electrophoresis buffer (1 mM Na_2_EDTA, 300 mM NaOH, pH 13). Electrophoresis was conducted for 30 min at 24 V (~0.74 V/cm) and 300 mA at 4°C. Then, the slides were immersed in neutralized buffer (0.4 M Tris-HCl, pH 7.5) and gently washed three times for 5 min at 4°C. All the above procedures were performed under dimmed light to prevent the occurrence of additional DNA damage. Comets were visualized by 80 *μ*L, 1X ethidium bromide staining (SigmaE-8751) and examined at 400 x magnification using a fluorescent microscope. Comet 5 image analysis software developed by Kinetic Imaging, Ltd. (Liverpool, UK) linked to a CCD camera was used to assess the quantitative and qualitative extent of DNA damage in the cells by measuring the length of DNA migration and the percentage of migrated DNA. Finally, the program calculates tail moment and Olive tail moment. In all the samples, 100 cells were analyzed and classified into 5 types (0–4) depending on their tail moment. Type 0 represents the cells without visible damage, while cells of type 4 have total degradation of DNA (long, broad tail, poorly visible head of the comet). Types 1, 2, and 3 represent the symptoms of increasing DNA damage. To calculate the extent of DNA damage, three types of the comet: numbers 2, 3, and 4 were selected.

### 2.5. Micronucleus Test

 Bone marrow slides for micronucleus assay from 5 mice of each group were prepared and stained according to the method described by Schmidt [21] using the modifications of Agarwal and Chauhan [22]. The bone marrow was flushed out from tibias using 1mL fetal calf serum and centrifuged at 2000 xg for 10 min. The supernatant was discarded. Evenly spread bone marrow smears were stained using the May-Grunwald and Giemsa protocol. Slides were scored at a magnification of 1000x using a light microscope. 1000 polychromatic erythrocytes per animal were scored, and the number of micronucleated polychromatic erythrocytes (MNPCE) was determined. In addition, the number of polychromatic erythrocytes (PCE) was counted in fields that contained 100 cells (mature and immature) to determine the score of PCE and normochromatic erythrocytes (NCE).

### 2.6. Mitotic Index Determination

Chromosomes were prepared according to the method described by Adler [23] with some modification. Briefly, 5 mice from control and treated groups were injected i.p with colchicine (2 mg/kg) 2 hours prior to tissue sampling. Bone marrow cells were collected from the tibia by flashing in KCl (0.075 M, at 37°C) and incubated at 37°C for 25 min. Material was centrifuged at 2000xg for 10 min, fixed in aceto-methanol (acetic-acid: ethanol. 1 : 3, v/v). Centrifugation and fixation (in the cold) were repeated five times at an interval of 20 min. The material was resuspended in a small volume of the fixative, dropped onto chilled slides, flame-dried, and stained the following day in 5% buffered Giemsa (pH 6.8). Slides were scored at a magnification of 1000x using a light microscope. At least 1000 cells were examined in each mouse and the number of dividing cells including late prophase and metaphase was determined. The mitotic activity is expressed by the mitotic index (MI), which is the number of dividing cells in 1000 cells per mouse.

### 2.7. Tumor Size Measurements

 Due to the high growth rate in Ehrlich tumor model, change in tumor volume (Δ*V*) was monitored over a period of 12 days for the four groups A, B, C, and D. Ellipsoidal tumor volume (*V*) was assessed and calculated using the formula *V* = (*π*/6)(*d*)^2^(*D*), where *D* and *d* are the long and short axes, respectively, measured with a digital caliper (accuracy 0.01 mm). Each data point was the average of 10 measurements taken every three days.

### 2.8. Statistical Analysis

Data were expressed as mean ± standard error. Statistical analysis was performed by one-way variance analysis ANOVA using SPSS (version 17.0). Difference were considered significant when *P* < 0.05. 

## 3. Results

**Figure 1:**
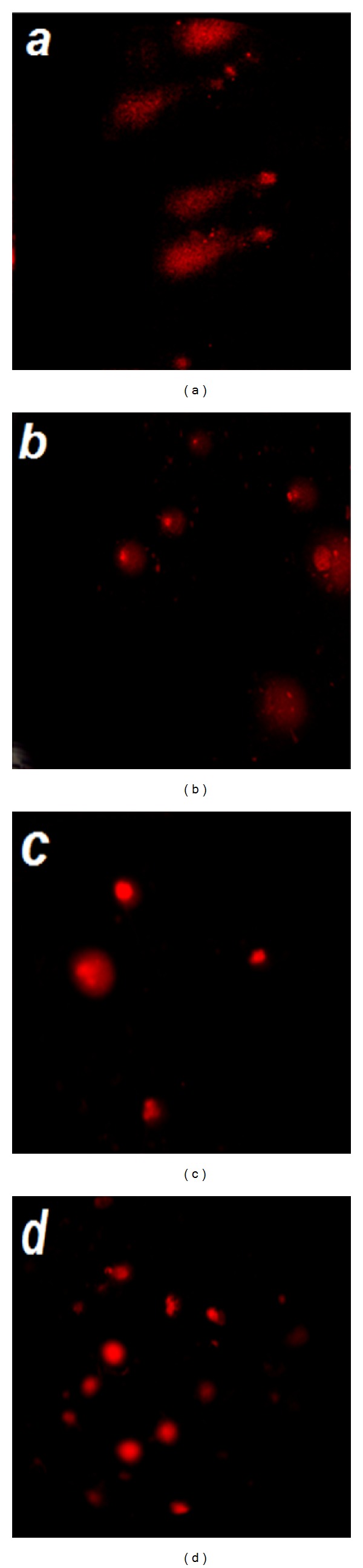
Typical comet images of Ehrlich carcinoma cells for (a) mice group (A) treated with cisplatin followed by exposure to ELF-MF, (b) mice group (B) treated with cisplatin, (c) mice group (C) treated by exposure to ELF-MF, and (d) mice group (D) the control one.

**Figure 2:**
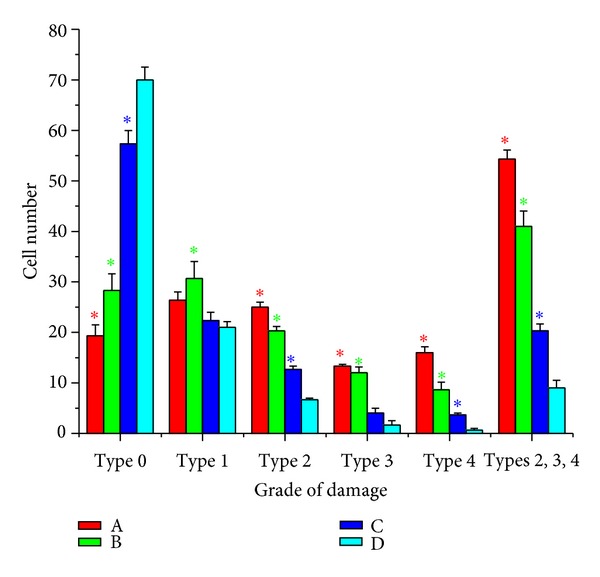
The level of DNA damage in Ehrlich tumor cells for mice group (A) treated with cisplatin followed by exposure to ELF-MF, mice group (B) treated with cisplatin, mice group (C) treated by exposure to ELF-MF, and mice group (D) the control one assessed by comet assay. Each value represents the mean ± SE (*n* = 5, *P < 0.001)

**Figure 3:**
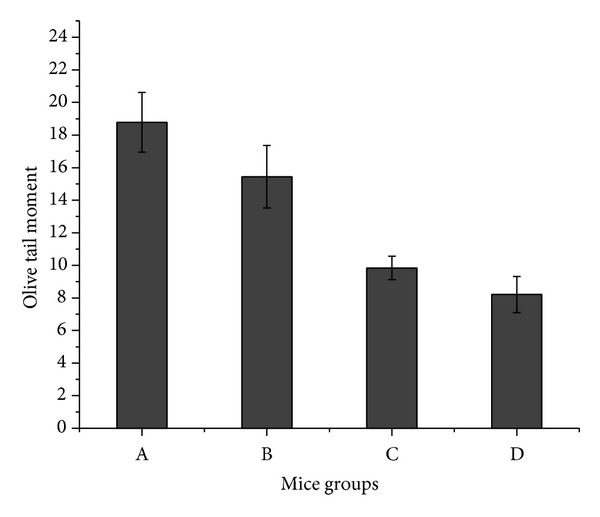
The values of olive tail moment assessed by comet assay for mice group (A) treated with cisplatin followed by exposure to ELF-MF, mice group (B) treated with cisplatin, mice group (C) treated by exposure to ELF-MF, and mice group (D) the control one. Each value represents the mean ± S.E. (*n* = 5, *P* < 0.019).

 The levels of DNA damage in cells of Ehrlich tumor showed a significant increase in treated groups (A, B, and C) compared to control group (D) (Figures 1 and 2). For type (0), the data revealed that about 70% of Ehrlich tumor cells did not exhibit any DNA damage in control group (D) compared to 19, 28 and 57% in treated groups A, B, and C, respectively. Meanwhile in type (4) about 16, 9 and 4% of Ehrilch tumor cells showed complete DNA damage in treated groups A, B, and C, respectively, relative to 1% for control group (D). The total percent of DNA damage in Ehrlich tumor cells represented by types (2, 3, and 4) showed five-, four- and twicefold increases for treated groups A, B, and C, respectively, with respect to control group (D). Also Figure 3 showed a significant increase (*P* < 0.019) in Olive tail moment for all treated groups compared to the control one.

Table 1 shows the frequencies of MNPCEs, PCEs, and NCEs in bone marrow cells of tumor bearing mice for both control group (D) and treated groups (A, B, and C). The results showed a significant increase in the formation of PCE for treated groups compared with that of control one. Also, MNPCEs induction showed an about 50% increase for treated groups (A and B) compared with the control group (D).

**Figure 4:**
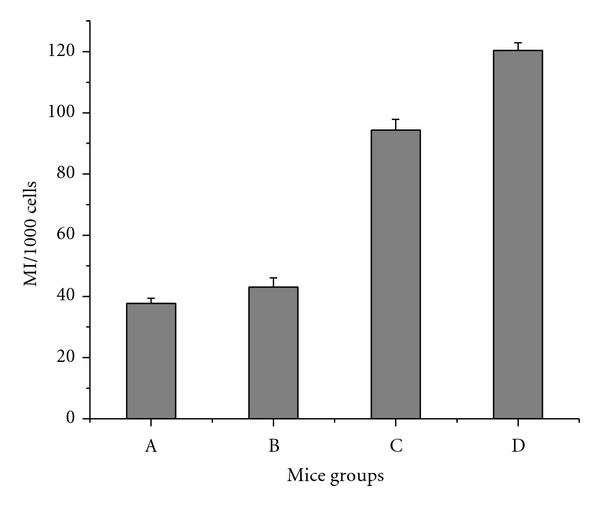
The values of mitotic index in bone marrow cells for mice group (A) treated with cisplatin followed by exposure to ELF-MF, mice group (B) treated with cisplatin, mice group (C) treated by exposure to ELF-MF, and mice group (D) the control one. Each value represents the mean ± S.E. (*P* < 0.001).

 The results of mitotic index (used to evaluate cell cycle kinetics) are summarized in (Figure 4. MI of bone marrow cells showed a significant decrease in the treated groups (A, B and C) (*P* < 0.001). The percent of decrement for the treated groups A, B, and C was about 75, 60, and 25%, respectively, in comparison with the control group (D). 

**Figure 5:**
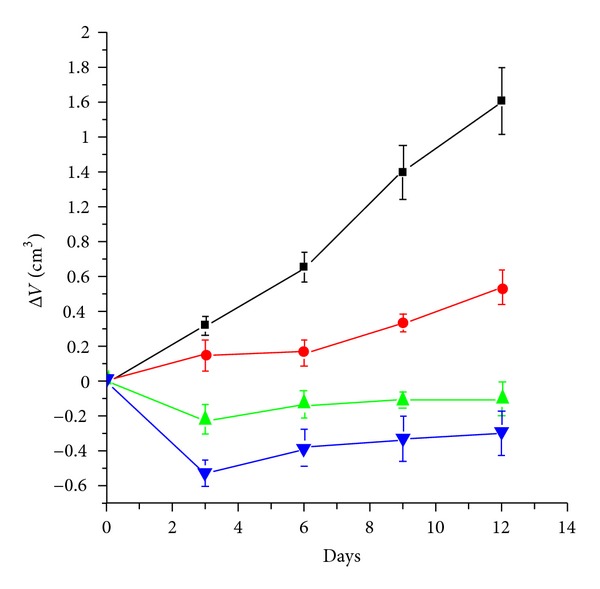
Average changes in Ehrlich tumor volume throughout a period of 12 days for mice group A (blue, (*▼*)) treated with cisplatin followed by exposure to ELF-MF, mice group B (green, (▲)) treated with cisplatin, mice group C (red, (•)) treated by exposure to ELF-MF, and mice group D (black, (■)) the control one. Each value represents the mean ± S.E. (*n* = 10).

Figure 5 shows the average change in tumor volume measured for mice of control group (D) and that of treated groups (A, B and C) over a period of 12 days. Under our experimental conditions, after 3 days, a significant decrease (*P* < 0.001) in tumor growth rate was observed in mice of groups A and B, while group (C) showed a slight delay in tumor growth rate (*P* < 0.049) compared with control group (D). The control group (D) showed a marked increase in tumor volume (growth) throughout the experimental time and the same behavior was observed for group (C), but with lower rate which is probably due to the existence of few viable tumor cells (Figure 5). The average tumor growth at day 12 for treated groups (A and B) was significantly less than that observed in control group (D) (*P* < 0.001).

Table 2 shows correlation coefficients between DNA damage, evaluated by comet assay, and cytogenic damage, measured by MN test. Both types of damage assessment are in good correlation, but the comet parameter, % DNA in tail, has a lower correlation with the MN values.

Table 3 shows correlation coefficients between the average change in Ehrlich tumor volume (Δ*V*) and MI. Both parameters are highly correlated. 

## 4. Discussion

 Cisplatin is one of the most widely used anticancer drug for the treatment of various cancers and solid tumors [24]. However, its major side effects are the main limiting factors of its clinical use for long-term treatment [9, 25]. Various treatment strategies and curing agents have been tried and used to monitor or control its side effects.

 Many anticancer agents exert their cellular toxicity through DNA damage [26], mainly DNA double-strand breaks. It is well known that DNA is the major target of cisplatin either as a result of its direct or indirect action through the generation of reactive oxygen species [10, 27–29]. 

 The current study had revealed that administration of low dose of cisplatin followed by ELF-MF exposure disrupts the integrity and the amount of intact DNA. Comet results emphasized the increase in the number of cells with damaged DNA types (2, 3, and 4) (Figures 1, 2, and 3) in treated group (A), this damage might be due to the involvement of free radicals even if their concentration has not yet been measured. Such observed DNA damage is in agreement with previous studies [30–32] who reported that cisplatin forms covalent platinum DNA adducts and also acts as a DNA alkylator. In addition, cisplatin generates reactive oxygen species, which trigger the opening of the mitochondrial permeability transition pore that permits the release of cytochrome c from mitochondria to cytosol and hence activates the mitochondria-dependent pathway leading to apoptosis [33, 34]. Also Tofani et al. [35] explained the synergistic activity observed between ELF-MF exposure and cisplatin by hypothesizing its ability to influence free radical chemistry exerted by the ELF-MF treatment.

 Micronucleus test is a very reliable, widely used assay to measure not only DNA damage but also chromosomal instability and cell death [36]. Our results showed that ELF-MF alone did not cause MN induction in bone marrow cells of tumor bearing mice, while treatment by both cisplatin combined with ELF-MF (group A) and cisplatin alone (group B) increased the induction of MN by about 50% compared to the control (group D) (Table 1). These results are in consistent with previous work by Miyakoshi et al. [37]. Moreover, correlation coefficients between MN test and the three measured parameters, determined by comet assay, pointed to a good relationship between DNA damage and MN induction induced by cisplatin and ELF-MF (Table 2). 

 The observed inhibition of mitotic index (Figure 4) in tumor-bearing mice bone marrow indicated that ELF-MF enhanced the cytotoxicity and genotoxicity of low dose cisplatin. These results were in good agreement with previous report on the genotoxic and cytotoxic potential of ELF-MF [13]. Moreover, the tumor growth suppression observed in treated groups A and B (Figure 5) and the high correlation between tumor growth inhibition and mitotic index (Table 3), emphasized that the treatment protocol used in this work is therapeutically beneficial as most likely enhanced the effectiveness of low dose cisplatin. The improvement in treated group A was superior to treatment with the same dose of cisplatin administrated to group B, which might be attributed to the increase in cell/tumor permeability induced by ELF- MF. 

 Consequently, our results indicated that increased damage of DNA by administrating low dose of cisplatin followed by ELF-MF exposure enhanced cell cytotoxicity as observed by the significant increase in micronucleus induction, in addition to a significant inhibition in both mitotic index and tumor growth. These results are in accordance with the commonly accepted assumption that extremely low frequency magnetic field enhanced the chemotherapeutic efficiency of cisplatin by increasing the production of oxygen species that caused more oxidative DNA damage.

## 5. Conclusion

 The data presented here seem to indicate that exposure to ELF-MF may be a useful adjunct to chemotherapy. However, further investigations are needed to optimize ELF-MF physical parameters, chemotherapy schedule, and combination of both.

## Figures and Tables

**Table 1:** PCEs, NCEs, MNPCEs induction in bone marrow cells of tumor bearing mice for control and treated groups.

Groups	NCEs/100	PCEs/100	MNPCEs/1000
A	54 ± 0.57*	46 ± 0.57*	11 ± 0.97*
B	57.3 ± 0.33*	42.7 ± 0.33*	11.0 ± 1.15*
C	65.3 ± 0.88*	34.7 ± 0.88	7 ± 1
D	65.3 ± 0.88	34.7 ± 0.88	7.3 ± 0.33

Mice group (A) treated with cisplatin followed by exposure to ELF-MF, mice group (B) treated with cisplatin, mice group (C) treated by exposure to ELF-MF and mice group (D) the control one. Each value represents the mean ± S.E. (*n* = 5, *P < 0.01).

**Table 2:** Correlation coefficients between DNA and MN.

	% DNA in Tail	Tail Length (*μ*m)	Tail Moment	Olive Moment
MN	0.446	0.649*	0.670*	0.738**

*P < 0.05, Person's correlation, **P < 0.01, Person's correlation.

**Table 3:** Correlation coefficients between MI and average change in Ehrlich tumor (*ΔV*).

	Average change in Ehrlich tumor (*ΔV*)
MI	0.808

*P* < 0.001, Person's correlation.
